# Current approaches to increase CAR T cell potency in solid tumors: targeting the tumor microenvironment

**DOI:** 10.1186/s40425-017-0230-9

**Published:** 2017-03-21

**Authors:** Irene Scarfò, Marcela V. Maus

**Affiliations:** Cellular Immunotherapy Program, Massachusetts General Hospital Cancer Center and Harvard Medical School, 149 13th Street, Room 7.219, Charlestown, Boston, MA 02129 USA

**Keywords:** Chimeric antigen receptor T-cells, Adoptive immunotherapy, Tumor microenvironment

## Abstract

Chimeric antigen receptor (CAR) T-cell therapy represents a revolutionary treatment for haematological malignancies (i.e. B-ALL). However, the success of this type of treatment has not yet been achieved in solid tumors. One hypothesis is that the immunosuppressive nature of the tumor microenvironment (TME) influences and affects the efficacy of adoptive immunotherapy. Understanding the role of the TME and its interaction with CAR T-cells is crucial to improve the potency of adoptive immunotherapy. In this review, we discuss the strategies and potential combinatorial approaches recently developed in mouse models to enhance the efficacy of CAR T-cells, with particular emphasis on the translational potential of these approaches.

## Background

**Fig. 1:**
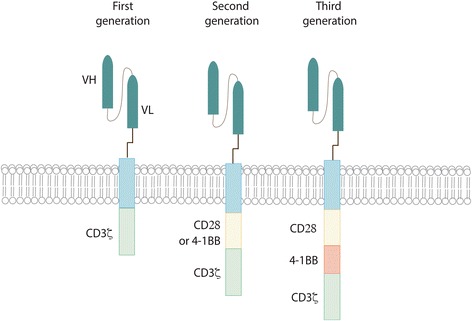
CAR structure. All different generations of CAR are composed of an extracellular antigen- binding domain (usually derived from an an antibody and engineered into an scFv), a hinge region, a transmembrane domain and various intracellular domains. First generation CARs have CD3ζ as the only signaling domain. In second generation CARs, one costimulatory domain was added, while third generation contain both CD28 and 4-1BB costimulatory signalling domains

Adoptive cell therapy (ACT) is a novel tool in the fight against cancer. In particular T-cells engineered to express Chimeric Antigen Receptors (CARs) have demonstrated recent significant clinical efficacy with improvements in patient outcomes for a number of hematological malignancies [1–4]. CARs are synthetic molecules composed of an extracellular binding domain, a transmembrane domain and an intracellular signaling/activation domain. The extracellular component consists of the light and heavy chain regions derived from an antibody to form a single chain variable fragment (scFv), and serves to recognize and bind specific tumor-associated antigens (TAAs) in a MHC-independent manner. A hinge domain, typically derived from CD8 or IgG4 molecules, connects this module with the intracellular one. This last portion is formed by CD3ζ segment which is responsible to trigger T-cell activation. The first generation of CAR vectors was designed with CD3ζ domain alone. Second and third generations added to CD3ζ one or two costimulatory domains (CD28 and/or 4-1BB) respectively (Fig. 1). All these components are typically inserted using γ-retroviral or lentiviral transduction systems. Although silencing of LTR-driven transgenes has been known to occur in other tissues, vector silencing was not observed in one study of human lymphocytes [5]. Interestingly, one study showed that efficacy of CAR T cells in vivo is a function of the density of CAR expression, and that this can have a substantial impact on antitumor efficacy and persistence of CAR T cells both systemically and at the tumor site [6].

**Fig. 2:**
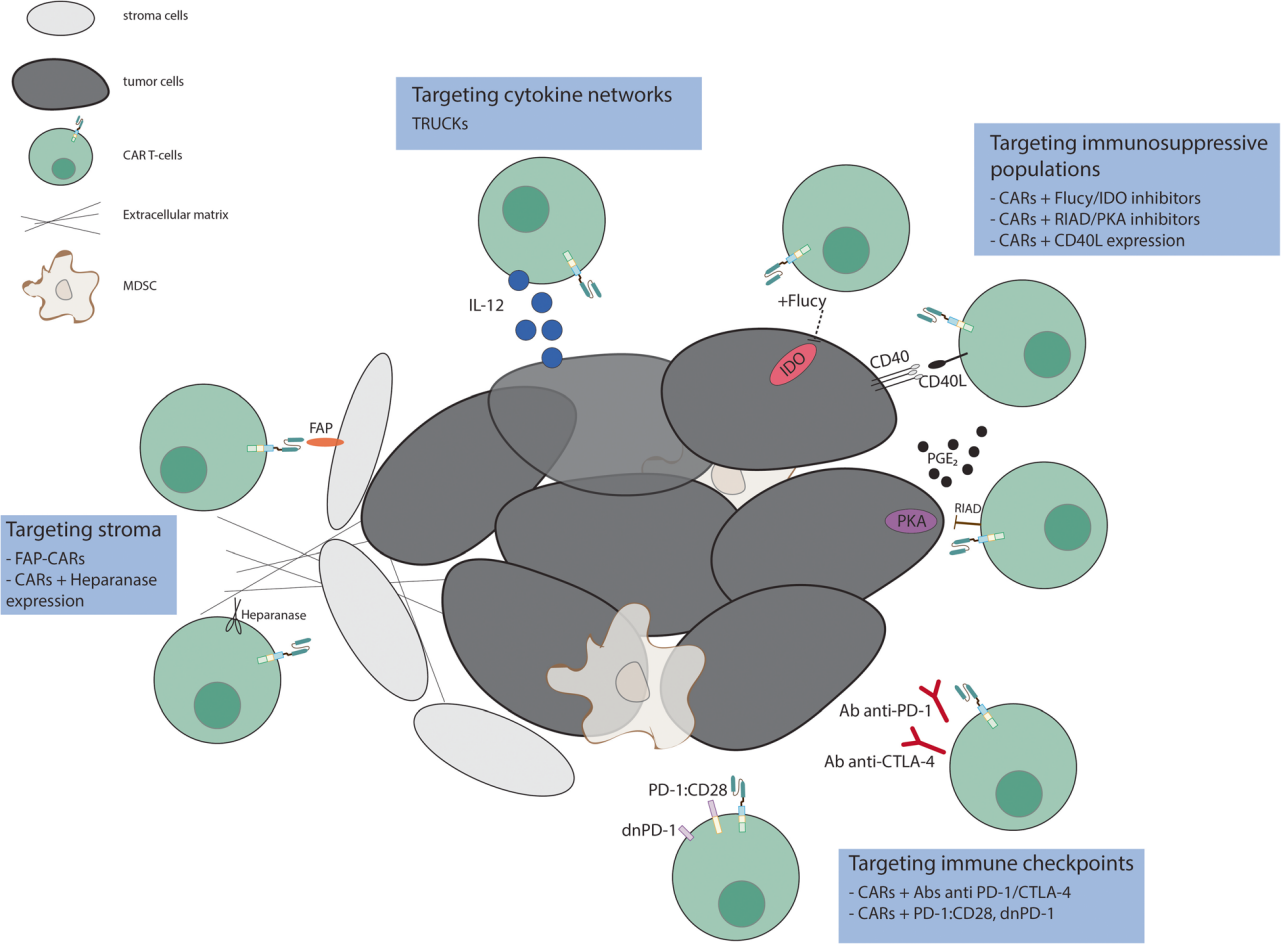
Targeting different components of the tumor microenvironment to enhance the efficacy of CAR T-cell therapy. Efforts to overcome the inhibitory effect of TME include strategies that target immunosuppressive populations (i.e. PGE_2_), stroma cells, cytokine networks and immune checkpoint signals

By combining the ability to avoid HLA restriction in antigen recognition with high specificity and potent activation, engineering these molecules to be expressed in T cells have emerged as one of the most promising approaches for cancer treatment. However, attempts to recapitulate the success achieved with CAR T-cells in B-cells malignancies for solid tumors has been disappointing. The three main hurdles encountered for the application of CAR T cell therapies to solid tumors are (1) the identification of proper tumor associated antigens, (2) the limited trafficking of adoptively transferred cells to tumor sites and (3) the immunosuppressive effect of tumor microenvironment. Here we will focus on approaches to address the third problem (Fig. 2); others have described approaches to the first two [7–13].

The complex and heterogeneous tumor microenvironment plays an essential role in tumor initiation progression and therapeutic resistance. Recent studies have highlighted the importance of innate immune activation for the generation of spontaneous T cell responses to tumor-associated antigens and anti anti-tumor activity Woo and colleagues showed that type I IFN-mediated activation of the STING pathway of cytosolic DNA sensing is one of the major players in sustaining a T cell inflamed-tumor phenotype which represents a favorable marker of responsiveness to immunotherapies [14]. Activation of the STING pathway contributes to activation of Batf3 dendritic cells, which appear to be central to anti-tumor immunity. These orchestrating dendritic cells are recruited by chemokines to the tumor site, then migrate to the secondary lymphatic organs and interact with CD8+ T cells. After clonal expantion, the T cells can migrate back to the tumor microenvironment and carry out their effector functions against tumor cells [15]. Although a thorough evaluation of innate immune activators and an inflamed-tumor microenvironment in the setting of CAR T-cells has yet to be investigated, the role of the tumor microenvironment in modulating efficacy of CAR-T cells is expected to be significant at the level of effector T cell function if not antigen presentation. Furthermore, the degree of intra-tumoral expansion and persistence that is required for clinical efficacy has not been determined for solid tumors. In this review, we discuss various aspects of the tumor microenvironment that could inhibit the efficacy of immune responses, and some of the approaches recently developed to reprogram the tumor microenvironment in order to enhance the efficacy of CAR T-cell therapy; some of these may also serve to enhance tumor sensitivity to other forms of immunotherapy. Indeed although the focus of this review is on CAR T cells, many of the principles herein discussed apply to and have been elucidated from studies of adoptive cell therapy more broadly, including tumor infiltrating lymphocytes (TILs) and TCR-engineered T cells [16–18].

## Targeting hypoxia, nutrient starvation, and metabolism

Hypoxia and nutrient starvation are major hallmarks of the tumor microenvironment [19]. The lack of nutrients, particularly amino acids such as tryptophan, is able to activate the integrated stress response which regulates T-cell activity [20]. Indoleamine 2,3 dioxygenase (IDO) is an intracellular enzyme which catalyzes the degradation of tryptophan to kynurenine. Both tumor cells and myeloid cells within the tumor microenvironment can express IDO, leading to a block in the proliferation and survival of T-cells. Recently, Ninomiya and colleagues [21] demonstrated that IDO expression can also inhibit CAR T cells. In particular, they showed that CD19 CAR T-cells fail to control progression of CD19+ IDO-expressing tumors. The accumulation of kynurenine inhibited expansion, cytotoxicity and cytokine secretion of CAR T-cells, suggesting a plausible mechanism underlying the resistance of IDO positive tumors to this type of treatment. Interestingly, they demonstrated that fludarabine and cyclophosphamide administration improved the efficacy of CAR T-cells by decreasing the expression of IDO. The combination of CAR T-cells and IDO inhibitors may represent a valuable option for malignancies resistant to chemotherapy and immunotherapy.

Moreover it is possible that metabolic stress in the tumor environment is able to modulate T cell metabolism, differentiation and effector functions. Indeed, tumor infiltrating lymphocytes modify their metabolism as a response to hypoxia, which is characteristic of solid tumors [22]. Interestingly, it has been demonstrated that CAR T-cells with different costimulatory domains utilize distinct metabolic pathways, which in turn reflect variable persistence within the tumor microenvironment [23]. Kawalekar and colleagues showed that 4-1BB CAR T-cells had increased mitochondrial biogenesis, which prompted a central memory phenotype and led to a survival advantage compared to CAR T cells with the CD28 signaling domain. In contrast, CD28-CAR T-cells yielded effector memory phenotype and enhanced glycolysis. This study highlighted the significance of the design of engineered T-cells with regard to their survival and exhaustion in the immunosuppressive tumor microenvironment.

Altering metabolic components within the tumor microenvironment is only one possibility for maximizing the potential of CAR T cells. In a more recent study Newick and colleagues [24] demonstrated similar findings by inhibiting protein kinase A (PKA) activation. PKA is the downstream effector of two other immunosuppressive factors produced within the tumor microenvironment: prostaglandin E2 (PGE_2_) and adenosine. Different studies have reported the role of these two molecules as potent inhibitors of T-cell proliferation and activity [25, 26]. The authors disrupted PKA anchorage to the lipid rafts by expressing RIAD (regulatory subunit I anchoring disruptor) peptide. This peptide is able to displace the association between PKA and ezrin, a protein necessary to tethering PKA close to adenylyl cyclase. Mesothelin-directed CAR-RIAD T-cells exhibited higher infiltration, persistence and antitumor activity in vivo compared to mesothelin-directed CAR T-cells. Moreover, the expression of RIAD led to an increased chemotaxis, guided by an induction of CXCR3 expression, and to better adhesion. As this approach is translated to clinical trials, the immunogenicity of the peptide may become an issue.

Another way to reprogram the tumor microenvironment is to augment the antitumor response of CAR T-cells by facilitating the recruitment of an endogenous immune response. This approach was validated by Curran and colleagues [27] through the constitutive expression of CD40L by CAR T-cells. They demonstrated that this strategy improves CAR T-cell cytotoxicity, decreases expression of PD-1, and improves DC antigen presentation function in a systemic model of CD40 positive follicular lymphoma. These mechanisms are likely to be synergistic with the STING pathways described by Gajewski and colleagues (as above).

Thus targeting tumor immunosuppressive populations such as PGE_2_ and/or T-cells response to the altered microenvironment represents an exciting opportunity to increase the efficacy of immunotherapy.

## Targeting stroma

Enhancing the efficacy of anti-tumor immune therapies could also be accomplished by targeting the non-malignant cancer-associated stromal cells (CASCs). These cells secrete growth factors, cytokines, and chemokines that promote tumor growth, metastasis and angiogenesis. One attractive stromal candidate is the fibroblast activation protein (FAP), a serine protease implicated in extracellular matrix remodeling and selectively expressed by CASCs in over 90% of epithelial cancers [28]. Three different groups have described the use of anti-FAP CAR T-cells with some contrasting results. Tran and colleagues [29] showed that despite anti-FAP CARs displaying specific degranulation and production of effector cytokines in response to antigen stimulation in vitro, they didn’t mediate an efficient antitumor response in vivo, and unexpectedly and alarmingly, anti-FAP CARs caused severe cachexia and lethal bone toxicities. The authors found that FAP protein is also expressed by multipotent bone marrow stromal cells (BMSCs) and related the observed toxicity to this expression. However, Kakarla and colleagues [30] using a FAP-CAR with a different scFv demonstrated antitumor efficacy without toxicities in a mouse model of human lung cancer. Even if T-cell persistence was limited, an improved response was achieved by co-injecting FAP-specific and tumor-specific T-cells. Schuberth and colleagues [31] developed an intra-peritoneal model for the adoptive transfer of FAP-CARs in a mesothelioma xenograft. Their data showed an increased survival; however, their anti-human FAP scFv did not have cross-reactivity with mouse FAP, which limited their ability to evaluate the on-target/off-tumor toxicity. A paper by Wang and colleagues [32] suggested that targeting FAP positive cells enhanced antitumor immunity via epitope spreading. They showed activation of endogenous CD4+ T-cells after three days of anti-FAP CAR treatment in an immune-competent syngeneic mouse model of mesothelioma and lung cancer. CD4+ activation was followed by a later augmentation of endogenous CD69+, INFγ + CD8+ T-cell infiltration. This antitumor response did not occur in immunodeficient mice, supporting the importance of the adaptive immune system. In another publication, the same group underlined the relevance of FAP inhibition by showing it also had an immune-independent antitumor effect. Using a weakly immunogenic and highly desmoplastic tumor, pancreatic ductal adenocarcinoma, Lo and colleagues [33] demonstrated that FAP-CAR T-cells inhibited tumor stromagenesis, reduced tumor vascular density and disrupted spatial orientation of tumor cells.

The safety concerns generated by the work of Rosenberg et al. [29] may be related to the specificity and affinity of the scFv, given that the last two studies with CAR T cells with different scFvs that recognize highly positive FAP cells, have been reassuring. Given the potential for multi-modal anti-tumor effects of FAP targeting, rational and interesting combinations for future immunotherapeutic approaches include anti-stroma CAR T-cells with either anti-tumor CAR T-cells or checkpoint blockade.

Beyond targeting FAP, another recent strategy for using CAR T cells in stroma-rich tumors is to target the enzyme heparanase (HPSE). This enzyme is responsible of the degradation of the heparan sulfate proteoglycans (HPGs), a fundamental process for the trafficking and accumulation of T-cells to the tumor site. Caruana and colleagues [34] demonstrated that CAR T-cells lose the expression of HSPE during their manufacturing process. This phenomenon leads to an impaired ability to degrade the extracellular matrix, suggesting a compromise in migration capacity. The authors showed that the induction of HSPE expression enhanced tumor infiltration and improved survival in neuroblastoma xenograft models.

## Targeting cytokine networks

Another potential option for shaping the tumor microenvironment to enhance ACT efficacy is to induce the local release of stimulatory factors that promote anti-tumor immune responses. In this context interleukin-12 (IL-12) and IL-18 represent promising candidates. In particular, IL-12 is an inflammatory cytokine, able to improve T-cell activation and induce a Th_1_ CD4+ T-cell response, CD8+ clonal expansion, and effector function. It is also able to recruit NK cells to the tumor site, reactivate anergic tumor-infiltrating lymphocytes (TILs), inhibit regulatory T-cells and the secretion of IL-10, IL-4 and trasforming growth factor beta (TGFβ) by tumor associated macrophages [35–39]. Starting from these considerations, several groups devised the “fourth generation” CAR T-cells combining IL-12 secretion with CAR expression. Koneru and colleagues [40] used the so called T-cells redirected for universal cytokine-mediated killing (TRUCK) to treat an orthotropic ovarian tumorgraft model. They showed complete eradication of tumor, prolonged persistence of CAR T-cells and higher systemic IFNγ levels. In order to increase the safety and reduce undesired toxicities that could be generated by constitutive, systemic high levels of IL-12, which have been toxic in clinical trials [41], they developed a tricistronic vector that encodes the scFv specific for MUC-16^ecto^ antigen, IL-12 and the truncated EGFR elimination gene (EGFRt) and administered it locally. Peritoneal administration of CAR T-cells was significantly more effective compared with intra-venous administration. This paper paved the way for a phase I clinical trial in platinum-resistant ovarian cancer patients, which will determine the safety and feasibility of this approach [42]. The production and release of a transgenic payload in a CAR should minimize toxicities in addition the “switch-off” system such as the EGFRt provides an additional measure of control for safety.

Furthermore IL-12 expression has proved to be important for the generation and efficacy of CAR T-cells from the umbilical cord blood (UCB). Pegram and colleagues [43] described a novel technique to expand and genetically modify UCB T-cells. First, they demonstrated that adding IL-12 and IL-15 to the cultured activated UCB T-cells led to a 150-fold expansion of this population, which showed an ideal phenotype expressing both memory and effector markers. Second, including the expression of IL-12 in the anti-CD19 CAR vector resulted in a significant increase in survival of mice bearing acute lymphoblastic leukemia (ALL), without the need for pretreatment or Il-2 support. These data support the clinical translation of using ACT to further boost the graft-versus-leukemia effect in UCB-transplanted patients with high-risk, relapsed/refractory ALL.

Another feasibile strategy to modulate tumor microenvironment signalling is to directly inhibit TGFβ, IL-10, and/or IL-4 signaling. The secretion of the first molecule by cancer cells and cancer associated cells is a well know mechanism of tumor evasion, and IL-10 as well as IL-4 are potent immunosuppressive cytokines. One approach is to force the overexpression of a dominant negative form of the receptor on T cells. Zhang and colleagues [44] demonstrated an increased antitumor efficacy in vitro and in vivo of T cells overexpressing TGFβ DNRII in a melanoma tumor model. Despite the promising results, further studies have to be done in orther to validate this approach in the context of CAR T-cell therapy. An additional recent system developed by Mohammed and colleagues [45] for the treatment of prostate cancer, a tumor characterized by elevated IL-4 levels, consists in an inverted cytokine receptor (ICR). Specifically, this 4/7 ICR is formed by the extracellular domain of the inhibitory IL-4 receptor linked to the intracellular immunostimulatory domain of IL-7. The co-expression of anti-PSCA first generation CAR vector and ICR resulted in an increased in vitro and in vivo antitumor activity. This approach transforms an inhibitory signal for T cells into a stimulatory one and at the same time deprives cancer cells of an important growth factor. The combination of a second generation CAR with 4/7 ICR could be evaluated to improve on these results.

## Targeting immune checkpoints

A major mechanism through which the tumor-microenvironment exerts its immune-inhibition is inducing the upregulation of surface inhibitory receptors such as cytotoxic T-lymphocyte-associate protein 4 (CTLA-4) and programmed death-1 (PD-1). These molecules are naturally upregulated after antigen-receptor engagement to dampen T-cell activation within tissues and maintain peripheral tolerance. A better understanding of the activation of these intrinsic inhibitory pathways by the tumor-microenvironment led to the success of immune checkpoint therapies [46]. Furthermore recent studies have uncovered the critical role of PD-1 in human CAR T-cell exhaustion. John and colleagues [47] published the first proof-of-concept study that CAR T-cells express PD-1 and are susceptible to PD-1 mediated suppression. The authors showed an improvement in antitumor activity when Her2+ tumor-bearing mice were treated with a combination of CAR T-cells and anti PD-1 antibody. Interestingly, the marked tumor regression was accompanied by a decrease of myeloid-derived suppressor cells (MDSC) in the tumor microenvironment. However, the mechanism of increased antitumor activity and role of the modulation of the MDSCs remain to be proven. A later study by Moon and colleagues [48] confirmed an augmented expression of PD-1 on CAR TILs which correlated with their hypofunction in a mesothelioma model. By blocking PD-1, they restored mesothelin-directed CAR T cell cytotoxicity in vitro. Beyond administering immune checkpoint antibodies, an alternative way of blocking immune checkpoints is to use genetic engineering strategies. For example, Liu and colleagues [49] inserted the PD1:CD28 switch-receptor into CAR vectors. This receptor was developed by Prosser et al [50] and it is engineered to express the extracellular domain of PD1 fused to the transmembrane and signaling domains of CD28; this construct could function as a dominant negative by competing with endogenous PD1 and/or could actively signal through the cytoplasmic domain after PD-L1 binding. The authors performed an analysis of effector functions of PD1:CD28 CAR T-cells injected intravenously to treat large, established, solid tumor including mesothelioma and prostate cancer in xenograft models. They reported a significant increase of the frequency of CAR TILs both in tumors and in peripheral blood, a greater ex vivo antitumor function, and more cytokine secretion. Of interest, the employment of a switch-receptor with a mutant signaling domain abrogated these results, suggesting a central role for the CD28 costimulatory domain in the fusion construct. Conversely, Cherkassky et al [51] demonstrated that combining CAR T-cells with the expression of a “dominant negative” form of PD-1 led to higher persistence, increased antitumor effects and prolonged survival in a mesothelioma xenograft model. The “dominant negative” form of PD1 in this model was composed of the only the extracellular domain of PD1 (without a signaling domain), which would presumably compete with endogenous PD1 for ligating PD-L1. One explanation for these divergent results could be the different type of tumor treated. Moreover, Cherkassky and colleagues showed that CAR T-cells with 4-IBB costimulatory domain were able to function at lower doses compared to CAR T cells that included CD28 signaling domains, and 4-1BB-signaling CARs were more resistant to PD-1 mediated exhaustion.

Additional tumor models are necessary to show that PD-1-mediated CAR T-cell suppression is a general inhibitory mechanism, particularly immunocompetent mouse models. It will be also important to understand the role of the costimulatory domains embedded into CARs and their differential mechanisms in mediating resistance to inhibitory molecules and inducers of CAR T-cell exhaustion. Even if the safest and most efficacius checkpoint combinations have yet to be identified [52], altogether these pre-clinical data provide supportive evidence that combining immune checkpoint blockade with CAR T-cells is a logical therapeutic strategy to improve the clinical outcome of CAR T-cell therapy in patients.

## Conclusions

Adoptive cell therapy using CAR T-cells has demonstrated impressive therapeutic potential for the treatment of certain B cell malignancies. Even though there have been recent exciting publications using TIL therapy targeting mutant KRAS metastatic colorectal cancer [53] and IL13Rα2-targeted CAR T cells in glioblastoma multiforme [54], results in solid malignancies may be subject to various limitations, including the immunosuppressive tumor microenvironment. Indeed, the microenvironment non only creates a physical barrier decreasing the penetration of modified T-cells into the tumor mass, but also plays an active role in immune suppression through the up regulation of inhibitory signals. Innovative strategies have been developed to overcome these challenges (Fig. 2), including co-administration of CAR T cells with checkpoint blockade, and co-administration with other drugs, therapies, and CAR T cells that target the tumor stroma and immunosuppressive molecules. Many of these strategies have been tested in xenograft and syngeneic mouse models, and clinical trials of these combinations are warranted and eagerly awaited. Pre-clinical experiments will define rational combinations of these approaches, based on a deeper understanding of the unique tumor characteristics and interplay among immune cells and the tumor environment. Translating the optimal combinations is likely to require iterative clinical trials to determine the safest and most effective combinations for patients with solid tumors.

## Abbreviations

ACT: Adoptive cell therapy
B-ALL: B-cells acute lymphoblastic leukemia
BMSCs: Bone marrow stromal cells
CAR: Chimeric antigen receptor
CASCs: Cancer-associated stromal cells
CD: Cluster of differentiation
CD40L: CD40 ligand
CTLA-4: Cytotoxic T-lymphocyte-associate protein 4
CXCR: C-X-C motif chemokine receptor
DC: Dendritic cell
EGFRt: Truncated epidermal growth factor receptor
FAP: Fibroblast activation protein
HLA: Human leukocyte antigen
HPGs: Heparan sulfate proteoglycans
HPSE: Heparanase
IDO: Indoleamine 2,3 dioxygenase
Ig: Immunoglobulin
IL: Interleukin
INFγ: Interferon gamma
MDSC: Myeloid-derived suppressor cells
MHC: Major histocompatibility complex
NK: Natural killer
PD-1: Programmed death-1
PD-L1: PD-1 ligand
PGE_2_: Prostaglandin E2
PKA: Protein kinase A
RIAD: Regulatory subunit I anchoring disruptor
scFv: Single chain variable fragment
TAAs: Tumor-associated antigens
TGFβ: Trasforming growth factor beta
TILs: Tumor-infiltrating lymphocytes
TME: Tumor microenvironment
TRUCK: T-cells redirected for universal cytokine-mediated killing
UCB: Umbilical cord blood
